# Variability in the rectus femoris muscle area and its association with clinical outcomes in critically ill patients: a prospective cohort study

**DOI:** 10.5935/0103-507X.20200023

**Published:** 2020

**Authors:** Laura Monteiro Magalhães, Eduardo Vestena Rossato, João Wilney Franco Filho, Wagner Luis Nedel

**Affiliations:** 1 Intensive Care Unit, Hospital São Lucas, Pontifícia Universidade Católica do Rio Grande do Sul - Porto Alegre (RS), Brazil.; 2 Intensive Care Unit, Hospital Ana Nery - Lajeado (RS), Brazil.; 3 Intensive Care Unit, Hospital Nossa Senhora da Conceição -Porto Alegre (RS), Brazil.; 4 Postgraduate Program in Biochemistry, Universidade Federal do Rio Grande do Sul - Porto Alegre (RS), Brazil.

**To the Editor,**

Muscle weakness is a common complication in critically ill patients, with loss of muscle mass during hospitalization in the intensive care unit (ICU), which is enhanced by the magnitude of organ failure and is associated with long-term morbidity.^(1,2)^ Rectus femoris muscle (RFM) ultrasound has emerged as a practical, easily performed and applied tool to monitor sarcopenia in patients at the bedside;^(3)^ however, its role in monitoring critically ill patients is uncertain. The main objective of the present study was to evaluate variations in RFM area throughout the ICU stay. The secondary objectives were to associate the variability in RFM area with ICU and hospital mortality; correlate the RFM area measurements with the Sequential Organ Failure Assessment (SOFA) on each measurement day; and evaluate the variation in area according to patient nutritional risk based on the Nutritional Risk in Critically Ill (NUTRIC) score.

We conducted a prospective and unicentric cohort study in a clinical-surgical ICU with 59 beds. Patients older than 18 years with acute dysfunction that motivated ICU hospitalization for a minimal of 3 expected days were included. The exclusion criteria were patients undergoing exclusive palliative care, with an expected ICU stay less than 3 days, and with risk of imminent death and amputees in whom measurement was not possible. Rectus femoris muscle ultrasound (Sonosite M-Turbo®, Sonosite Fuji Film, Bothell, USA) was performed using a previously described methodology.^(4)^ We performed measurements on the first (A1), third (A3) and tenth (A10) days of ICU stay by 2 previously trained evaluators. Clinical variables, comorbidities, Simplified Acute Physiology Score (SAPS 3), SOFA and NUTRIC were analyzed. The project was approved by the Research Ethics Committee of *Grupo Hospitalar Conceição* (62104416.1.0000.5530). A free and informed consent form was signed by the study participants or their legal guardian. Associations between categorical variables were tested using the chi-square test. The comparison of continuous variables between groups was performed using Student’s *t* test or the Mann-Whitney test. The area differences at the different time intervals were analyzed using the Wilcoxon-signed rank test with Bonferroni correction. The correlation between SOFA variability and the variability in area at the different intervals was evaluated using the Pearson correlation test. We assumed an alpha error of 5%.

**Figure 1:**
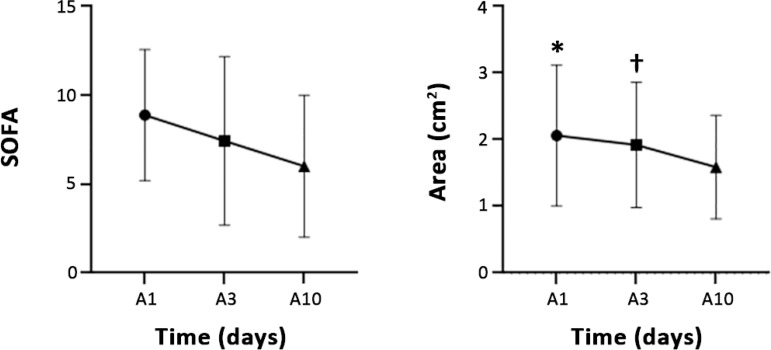
Variation in area and Sequential Organ Failure Assessment score during hospitalization in an intensive care unit. SOFA - Sequential Organ Failure Assessment; A1 - rectus femoris muscle area on the first day of admission; A3 - rectus femoris muscle area on the third day of admission; A10 - rectus femoris muscle area on the tenth day of admission. * p < 0.0001 between A1 and A10; † p < 0.005 between A3 and A10.

A total of 69 patients were included, of whom 31 (44%) also underwent a 10^th^-day measurement. The sample consisted mostly of women (60%), 75% of the population presented high nutritional risk according to the NUTRIC score, and 71% of patients had sepsis. The mean SAPS 3 score was 73.2 points, and the SOFA score on admission was 8.8 (± 3.6) points. The mean length of stay in the ICU was 15.5 days, and the mean hospital stay was 40.5 days. The mortality rate in the ICU was 44.9%, and the hospital mortality rate was 55%. There was a statistically significant decrease in RFM area during ICU admission - A1, 1.74 (1.38 - 2.29); A3, 1.6 (1.31 - 2.28), and A10, 1.05 (1.05 - 1.81); p < 0.0001 -, with a difference between A1 and A10 (p < 0.0001) and between A3 and A10 (p < 0.005) but not between A1 and A3 (unadjusted p = 0.25) (Figure 1). The delta A1-A3 did not correlate with the SOFA variation measured on the same days, with a Pearson coefficient of 0.07 (p = 0.35). The variability between A1 and A3 was not different between the high and low nutritional risk groups (-2.51 ± 19.82 in the high-risk group and -4.55 ± 20.03 in the low-risk group; p = 0,231). The variability between A1 and A10 was not different between the groups (-8.27 ± 20.83 in the high-risk group and -14.94 ± 39.39 in the low-risk group; p = 0.478). We established a cut-off point of 10% RFM area reduction as clinically relevant, and patients with a reduction ≥ 10% did not have a SAPS 3 score at admission higher than that of patients with a reduction of < 10%, both between A1 and A3 (74 ± 13 versus 71 ± 16; p = 0.40) and A3 - A10 (78 ± 16 versus 78 ± 13; p = 0.77). At this cut-off point for area loss, the SOFA score at admission did not differ between A1 - A3 (8 ± 4 versus 9 ± 3, p = 0.14, in the groups with area loss ≥ 10% and in the other patients) as well as between A3 and A10 (8 ± 3 versus 10 ± 3, p = 0.25, in the groups with area loss ≥ 10% and in the other patients). Other outcomes are presented in table 1.

**Table 1:** Correlation between rectus femoris muscle area and clinically relevant outcomes

Variable	Mean survivors	Mean non-survivors	Mean difference (95%CI)
Delta A1 - A3	-4.72	-1.05	-3.6 (-16.1 - 8.8; p = 0.56)
Delta A1 - A10	-21.12	-14.1	-7.0 (-21.1 - 7.1; p = 0.31)
	Variation ≥ 10% between A1 and A3	Variation < 10 % between A1 and A3	p
MV time	9 ± 13	7.5 ± 14	0.7
Length of ICU stay	10 ± 14	10 ± 16	0.49
Length of hospital stay	27 ± 29	33 ± 38	0.3

95%CI - 95% confidence interval; A1 - rectus femoris muscle area on the first day of admission; A3 - rectus femoris muscle area on the third day of admission; A10 - rectus femoris muscle area on the tenth day of admission; MV - mechanical ventilation; ICU - intensive care unit.

Although we found a significant reduction in RFM area, it was not associated with changes in clinically relevant outcomes, unlike in previous studies, which were also from a single center. This may be partially explained by the fact that our population had a higher severity profile when evaluated by the SAPS 3 and SOFA.^(2,5)^ In addition, our sample consisted of patients with a high prevalence of high nutritional risk. In these clinical scenarios, the variability in RFM has not yet been adequately studied; therefore, we cannot infer that this variable has the same behavior as that in a population with less severe or low nutritional risk, although patients with high nutritional risk have not shown higher muscle loss. Patients with more severe muscle loss (≥ 10%) presented the same severity at admission to the ICU as did the other patients. Although muscle loss may be associated with increased inflammatory activity, which in turn is associated with greater severity of the patient upon admission to the ICU,^(1)^ we did not find a relationship between initial severity (measured by the SAPS 3) and greater muscle loss intensity in our sample. Therefore, the global assessment of skeletal musculature and, in particular, the variability in RFM area should be more widely studied in the most critical disease scenarios, preferentially through multicenter studies with large sample sizes, with qualitative and quantitative measurements, establishing their role in monitoring and prognosis during the evolution of critical illness.
